# A field comparison study of two vaccine protocols against *Erysipelothrix rhusiopathiae* in two types of swine breeds in Spain

**DOI:** 10.1186/s12917-024-04065-0

**Published:** 2024-10-11

**Authors:** E. Sanchez-Tarifa, C. Alonso, I. Perez, L. A. García, A. Fernández-Fontelo, O. Gómez-Duran, B. García-Morante, Francisco A. García–Vázquez, I. Hernández-Caravaca

**Affiliations:** 1 Boehringer Ingelheim Animal Health, Sant Cugat del Vallès, Barcelona, Spain; 2 Boehringer Ingelheim Vetmedica GmbH, AH Swine, Ingelheim, Germany; 3 Inga Food S.A., Tres Cantos, Spain; 4 Alvettia Gestión y Control S.L., Talavera de la Reina, Spain; 5 School of Business and Economics, Humboldt-Universität zu Berlin, Berlin, Germany; 6 Departament de Matemàtiques, Universitat Autònoma de Barcelona, Barcelona, Spain; 7 Centcinc, C/Montserrat de Casanovas 105, Barcelona, 08032 Spain; 8 Department of Physiology, Faculty of Veterinary Science, University of Murcia, Murcia, Spain; 9 Department of Community Nursing, Preventive Medicine and Public Health and History of Science, University of Alicante, Campus de Sant Vicent del Raspeig. Ap. 99, Alicante, E-03080 Spain

**Keywords:** Swine erisipelas, Sow vaccination, Maternally derived antibodies (MDA), ELISA

## Abstract

Erysipelas still causes large economic losses to pig industry. Maternal immunity is critical to prevent erysipelas in young animals, thus, intensive vaccination protocols or practices focused on the improvement of the maternally derived immunity could provide substantial benefits. The present study evaluates potential changes in antibodies levels in sows and their offspring using two types of tests (commercial ELISA, Ingenasa or rSpaA415 ELISA) when two different vaccination programs (before farrowing or after farrowing) against *Erysipelothrix rhusiopathiae* were applied to sows from Iberian (A) or conventional Large White-Landrace (B) pig farms. The results showed a statistical correlation between titers found in sows and their one-week old piglets in both tests. The overall mean of (log) antibody titers in farm B measured by the commercial ELISA test was significantly higher in pre-farrowing vaccinated sows compared to the post-farrowing vaccine protocol (*p* = 0.0278). Additionally, using the rSpaA415 ELISA test, the overall mean of (log) antibody titers was significantly higher in pre-farrowing sows (*p* = 0.0056) compared to sows following post-farrowing vaccine protocol (*p* = 0.0003) or non- vaccinated sows. None of the above-mentioned differences were found in farm A. The overall mean of (log) antibody titers in piglets from the pre-farrowing vaccination protocol was significantly higher than piglets from the post-farrowing vaccination protocol in farm A (*p* = 0.0059; rSpaA415 ELISA) and farm B (*p* = 0.0168 and *p* = 0.0098 for the commercial and rSpaA415 ELISA data, respectively). Additionally, higher proportion of piglets from pre-farrowing vaccinated sows remained seropositive during the post-weaning period (days 42 to 84) compared to piglets from non-vaccinated or post-farrowing vaccinated groups in both farms A and B.

**Supplementary Information:**

The online version contains supplementary material available at 10.1186/s12917-024-04065-0.

## Introduction

The infectious disease caused by *Erysipelothrix* spp. is one of the oldest recognized diseases that affect growing and adult swine. Erysipelas causes large economic losses due to outbreaks of acute septicemia, with or without cutaneous lesions, abortions or increased pre- and postpartum vulvar discharge, as well as smaller litter sizes and reduced numbers of live born piglets in breeding sows or chronic infections causing polyarthritis and/or proliferative endocarditis [1, 2]. There are four *Erysipelothrix* spp. and 28 different serotypes described thus far. However, *Erysipelothrix rhusiopathiae (E. rhusiopathiae*) serotypes 1a, 1b, and 2 are the most commonly isolated from clinically affected pigs and have the highest prevalence and economic impact [3, 4]. Despite being well-controlled for decades through vaccination, several studies of swine erysipelas outbreaks in countries with relevant pig production have raised concerns on disease re-emergence [5–9]. Thus, it is still of critical importance to revisit the currently available control strategies against erysipelas and gain prospects for the future.

*E. rhusiopathiae* is worldwide in distribution and ubiquitous, and pigs are considered the most important reservoir. It is estimated that 30-50% of healthy pigs harbour the organism in their lymphoid organs, especially in the tonsils [10]. Carriers and pigs with acute erysipelas can shed the bacterium in their urine, faeces, and respiratory secretions for extended periods of time, enhancing transmission and a relatively high prevalence of infection [2, 11]. Indeed, contamination of the environment by carriers with no symptoms is thought to be one of the main causes of disease transmission [2, 11]. Considering the high risk of erysipelas, vaccination is worthwhile [11, 12]. In pigs, live attenuated vaccines or bacterins are frequently used, and regardless of the vaccine type, erysipelas vaccines are considered the most efficient and practical way of preventing the disease [11]. While vaccination against *E. rhusiopathiae* at every breeding cycle is standard in most pig breeding herds, growing pigs are not vaccinated as commonly. However, fattening pigs might be vaccinated if there is a perceived high risk of erysipelas. For instance, straw based, and outdoor production systems such as Iberian production, may predispose to a higher risk of suffering the disease [11]. In these cases, intensive vaccination protocols or practices focused on the enhancement of the maternally derived immunity could provide substantial benefits.

In erysipelas, antibodies against *E. rhusiopathiae* are known to play an important role in protection [13, 14]. Protective immunity after vaccination is generally thought to range from 4 to 6 months [11], whereas passively acquired antibodies have been detected in weaned pigs up to 8 weeks of age [15]. Several serological methods for diagnosing chronic swine erysipelas or assaying maternally derived antibodies (MDA) and acquired antibodies before and after vaccination have been reported [16, 17]. Even though the poor sensitivity of the currently available enzyme-linked immunosorbent assay (ELISA) tests has limited their widespread use for evaluation of exposure to *E. rhusiopathiae*, ELISA is the test of choice among existing serological procedures because it is simple, allows the testing of a large number of samples in a short period of time, and provides concise and objective results [17].

Maternal immunity is critical to prevent erysipelas in young animals [15]. There is, however, a small number of research studies focusing on alternative vaccination protocols in sows that may increase protection provided by MDA in piglets via the colostrum [18]. Therefore, the present study evaluates potential changes in antibody levels in sows and their offspring when two different vaccination programs against *E. rhusiopathiae* were applied to sows of conventional Large White-Landrace farms and Iberian pig farms. In particular, the objectives of the current study are three-fold: (1) to study the evolution of seroconversion over time, as well as the time-to-negative status between protocols in both sows and piglets, (2) to assess the antibody level dynamics in sows and piglets from the two types of swine farms studied, and (3) to investigate the correlation between E. rhusiopathiae ELISA titers in sows and their offspring, as well as the concordance between the ELISA tests used. Henceforth, the present study can be viewed as the first step in trying to improve maternally derived immunity on piglets against *E. rhusiopathiae* via alternative vaccination protocols in breeding sows.

## Materials and methods

### Farms description

The study was conducted on two sow farms. Farm A consisted of a 900-sow farrow-to-nursery Iberian production system combining outdoor and indoor conditions, whereas Farm B comprised 1,800-sows in a farrow-to-wean intensive conventional system with Large White-Landrace genetics. Both farms were in Spain’s central area, with an industry standard biosecurity program in place certified by the herd veterinarian. The sows on farms were actively vaccinating against the porcine respiratory and reproductive syndrome virus, Parvovirus and *E*. *rhusiopathiae*.

Regarding swine erysipelas, gilts from Farm A were vaccinated intramuscularly (i.m.) at 12 and 16 weeks of age with 2 mL of Eryseng® (HIPRA, Amer, Spain), and at 32 and 36 weeks of age with 2 mL of Eryseng® Parvo .A re-vaccination protocol of the multiparous sows was followed by applying 2 mL of Eryseng® Parvo 10 days after farrowing. In addition, piglets from farm A were vaccinated i.m. with 2mL of Eryseng® at 12, 16, and 32 weeks of age following the Iberian pig industry standards. In farm B, gilts were vaccinated i.m. at 22 and 26 weeks of age with 2 mL of Eryseng® Parvo followed by a re-vaccination protocol of the multiparous sows with 2 mL of Eryseng® Parvo 10 days after farrowing.

### Study design

Table 1 shows the vaccination protocol groups and the number of pigs assigned to each group in farms A and B. On farms A and B, 24 and 35 sows (of the same batch), respectively, were assigned into five groups according to the parity number, type of erysipelas vaccine, and vaccination protocol (pre-farrowing, post-farrowing and control). Parity distribution was balanced among gilts and multiparous sows on each group in order to be considered in the analysis. A negative control group without vaccination (non-vaccinated) was also included. Two commercially available vaccines were used in this study. Approximately 30 days before farrowing (pre-farrowing) or 10 days after farrowing (post-farrowing), 2 mL of each vaccine were administered i.m. in one side of the neck. Sows in groups 1 and 3 were vaccinated with Ruvax® (Boehringer Ingelheim Vetmedica Inc., St. Joseph, MO, USA), whereas sows from groups 2 and 4 were vaccinated with Eryseng®. The immunization protocols used agreed with the manufacturer’s recommendations in both cases.

**Table 1:** Study design. A total of 24 and 35 sows were randomly assigned to a treatment group in farm A and B, respectively. Treatment groups were established based on the type of commercial vaccine and the immunization protocol applied

Farm/Breed	Group (Number of sows)	Vaccine	Vaccination Protocol (Day of vaccination)
A/Iberian	1 (*n* = 6)	Ruvax®^1^	Pre-farrowing (-30)
	2 (*n* = 6)	Eryseng®^2^	
	3 (*n* = 4)	Ruvax®	Post-farrowing (+ 10)
	4 (*n* = 4)	Eryseng®	
	5 (*n* = 4)	Control	Non-vaccinated
B/Ld-Lw	1 (*n* = 7)	Ruvax®	Pre-farrowing (-30)
	2 (*n* = 7)	Eryseng®	
	3 (*n* = 7)	Ruvax®	Post-farrowing (+ 10)
	4 (*n* = 7)	Eryseng®	
	5 (*n* = 7)	Control	Non-vaccinated

^1^Boehringer Ingelheim Vetmedica Inc., St. Joseph, MO, USA

^2^HIPRA, Amer, Spain

**Fig. 1:**
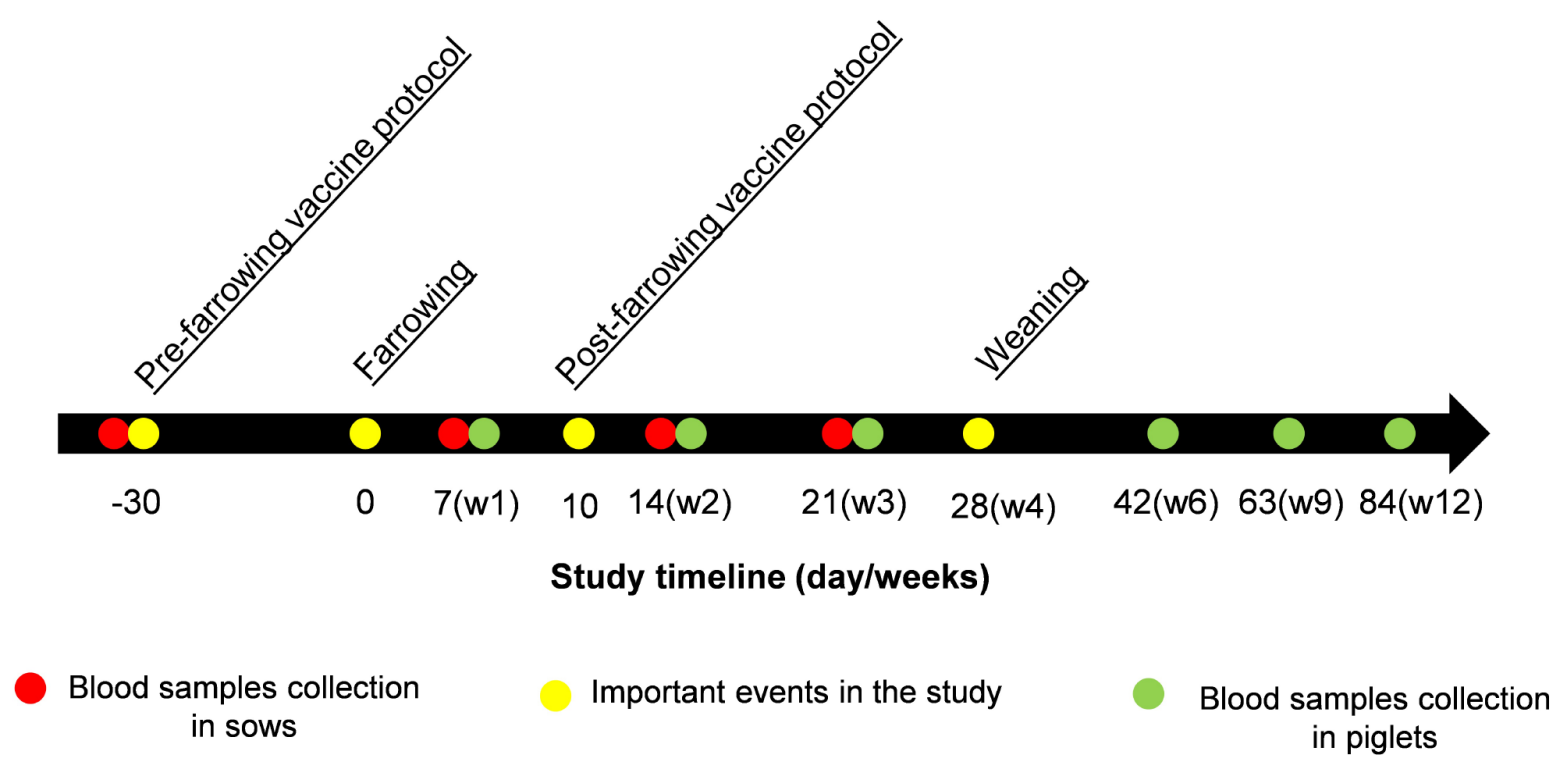
Timeline of sampling and important events in the study. Red and green circles illustrate blood sample collection in sows and piglets, respectively. Yellow circles highlight vaccine and management events in the study

Blood samples from all sows and two randomly selected piglets from each litter were collected (48 and 68 piglets from farms A and B, respectively). The piglets were able to received colostrum from their own mom as split-nursing was applied if required [19]. The piglets included in the study were ear-tagged at one week of age to help with their identification later on. Blood samples were obtained from the jugular vein at different time points, with the farrowing day serving as the study day 0 (Fig. 1). Blood was obtained from sows before vaccine administration at -30 ± 5 days (i.e., 80–85 days of gestation) and weekly after farrowing at 7, 14 and 21 days. From piglets, blood was obtained weekly after birth at days 7, 14, and 21, and after weaning at days 42 and 63 in both farms. An additional sampling was taken at 84 days of age in farm B (Fig. 1). Piglets on farm A were only monitored until day 63 because they were vaccinated against erysipelas at the beginning of the finishing period.

### Blood processing and serology tests

All the blood samples were centrifuged to separate the serum, and serum samples were aliquoted into appropriate tubes and kept at either 2–8 °C or -20 °C (± 5 °C) until testing could be performed. Sera were tested for *E. rhusiopathiae* antibodies using a commercial ELISA (Ingezim® Mal Rojo, INGENASA, Madrid, Spain) and a previously described ELISA based on a recombinant SpaA protein (rSpaA415; Giménez-Lirola et al., 2012). The commercial ELISA performance was self-verified, and it was used and interpreted according to the manufacturer’s recommendations. Sensitivity and specificity (98.4% and 89.09% respectively) of this ELISA was calculated by comparison with the reference technique (slow agglutination). Briefly, the positive cut-off was set at the negative control’s optical density (OD) + 0.200, and any sample with an OD value greater than this, cut-off was declared positive. For titration purposes, the titer of each sample was the maximum dilution that produced a positive result. The rSpaA415 ELISA was performed and read according to the protocol described by Giménez-Lirola et al. (2012). A sample was considered positive if the OD value exceeded 0.9, with an overall sensitivity and specificity of 96.5% and 100%, respectively [17]. The objective of using both tests is to achieve greater robustness of the results obtained by applying different methods to evaluate antibody levels and seroconversion.

### Statistical analysis

First, a collection of graphs was created to provide a concise description of our data. In particular, scatterplots were used to describe the evolution of antibody titers over time for each of the vaccination protocols for either commercial ELISA or rSpaA415 ELISA tests. Also, bar plots were considered to depict the proportion of positive and negative individuals at each time point according to vaccination protocol and ELISA test. Also, Chi-square test was used for comparison between proportions of seropositive and seronegative animals (sows and piglets) within the three protocols (pre-farrowing, non-vaccinated, post-farrowing) and type of analysis (commercial vs. rSpaA415 ELISA test) used at the different time points. The results are presented as percentages (%), and differences were considered statistically significant when *p* < 0.05. Survival curve plots were created to visually compare the estimated survival distributions among vaccination protocols and ELISA tests in sows.

Second, two types of methods were considered for statistical analysis and inferences: For the first objective, it was used survival analysis, particularly the Log Rank test [20] to compare the time to seroconversion curves of pre-farrowing and post-farrowing protocols, and the Cox-Hazard Proportional Model [20] to estimate the differences in such curves between pre-farrowing and post-farrowing protocols if statistical differences were previously found in the Log Rank test. It was considered using survival analysis to address this current paper’s first objective since times to seroconversion revealed right-censoring.

For the second objective, a linear mixed model (LMM) [21] was used with fixed effects such as the protocol vaccination type and random effects such as the sows’ or piglets’ intrinsic variability as we measured antibody titers within the same individual at different time points and thus measures within the same individual could potentially be highly correlated. The (log) antibody titers (response variable) was regressed onto a collection of covariates acting as fixed effects in the model: it was included as covariates the (log) antibody titers at the beginning of the study to control for baseline heterogeneities unrelated to the treatment effect (only for sows), the protocol (primary covariate) as a factor with three levels “pre-farrowing”, “post-farrowing” and “non-vaccinated” (baseline), the time points when we measured antibody titers specified as a factor with levels differing between sows and the age of sows specified as a factor with levels “gilt” or “multiparous”. Two interactions were further considered between some of the covariates described above: The interaction between protocol and time to evaluate differences in (log) antibody titer means at each time point between pre-farrowing, post-farrowing and non-vaccinated protocol groups, and the interaction between protocol, time, and sows age to investigate whether (log) antibody titer means between protocols at different time points were different depending on the age of sows. Sows or piglets were included either as a random effect in the intercept of the corresponding model to control for intra-sows or intra-piglets variability as our response variable was measured over time, and thus, measures within the same individual were likely to be correlated. Finally, note that it was considered log-transforming the response variable (i.e., antibody titers) to correct for asymmetry in its empirical distribution as LMM assumes a normally - thus symmetrically - distributed response variable.

Both data description and statistical analysis and inferences were done with the R software via the R Studio interface (versions R 3.6.0, R Studio 1.2.1335). Data description was conducted with libraries base, ggplot2 and survmine, survival analysis with library survival, and finally, the LMM model was estimated with library name and means comparison was performed with library emmeans.

For the third objective, one week post farrowing sows and their 1-week-old piglets of the two farms were analyzed together by linear regression for both tests, fitting the requirements of model adequacy checking. Kappa statistic was calculated for paired tests using dichotomized data for both farms and tests. For assays in which results were identified as positive or negative (commercial ELISA and rSpaA415 ELISA). Values for kappa range from − 1 to 1 where − 1 indicates agreement worse than expected by chance, 0 equals agreement no better than expected by chance and 1 equals complete agreement [22]. The following arbitrary standards for the strength of agreement as described by Landis and Koch were used: 0 ≤ poor, 0.01–0.2 = slight, 0.21–0.4 = fair, 0.41–0.60 = moderate, 0.61–0.80 = substantial and 0.81–1 = almost complete [23]. All statistical analyses of this objective were performed using Minitab^®^ version 20.2 (Minitab^®^, LLC: State College, PA).

## Results

### Serology in sows

Sows from farms A (Iberic) and B (Large White-Landrace) were distributed into five groups considering the vaccine type (i.e., Ruvax® vs. Eryseng®) and the vaccination protocol (i.e., pre-farrowing, post-farrowing and control) as shown in Table 1. All the sows that started the study remained to the end, except one (sudden death) in farm B assigned to the post-farrowing vaccination protocol and inoculated with Eryseng®.

Blood samples were collected at different sampling time points across the study (Fig. 1), and sera were tested for *E. rhusiopathiae* antibodies using a commercial ELISA (Ingezim® Mal Rojo, INGENASA, Madrid, Spain) and a previously described ELISA based on a recombinant SpaA protein (rSpaA415; Giménez-Lirola et al., 2012).

As no difference were found regarding the vaccine type in terms of titers, and percentages of seropositive results, the data from both vaccines are shown aggregated onwards. Sows from farm A (Iberic) had remarkably high antibody titers across all the study sampling points. Given that all of them remained seropositive throughout the study period, no statistical analysis was conducted to investigate the relationship between the evolution of seroconversion over time and other variables, e.g., vaccination protocol, thus the analyses explained below refer exclusively to sows from farm B.

*Objective 1. Seroconversion evolution over time and time-to-negative status*

**Fig. 2:**
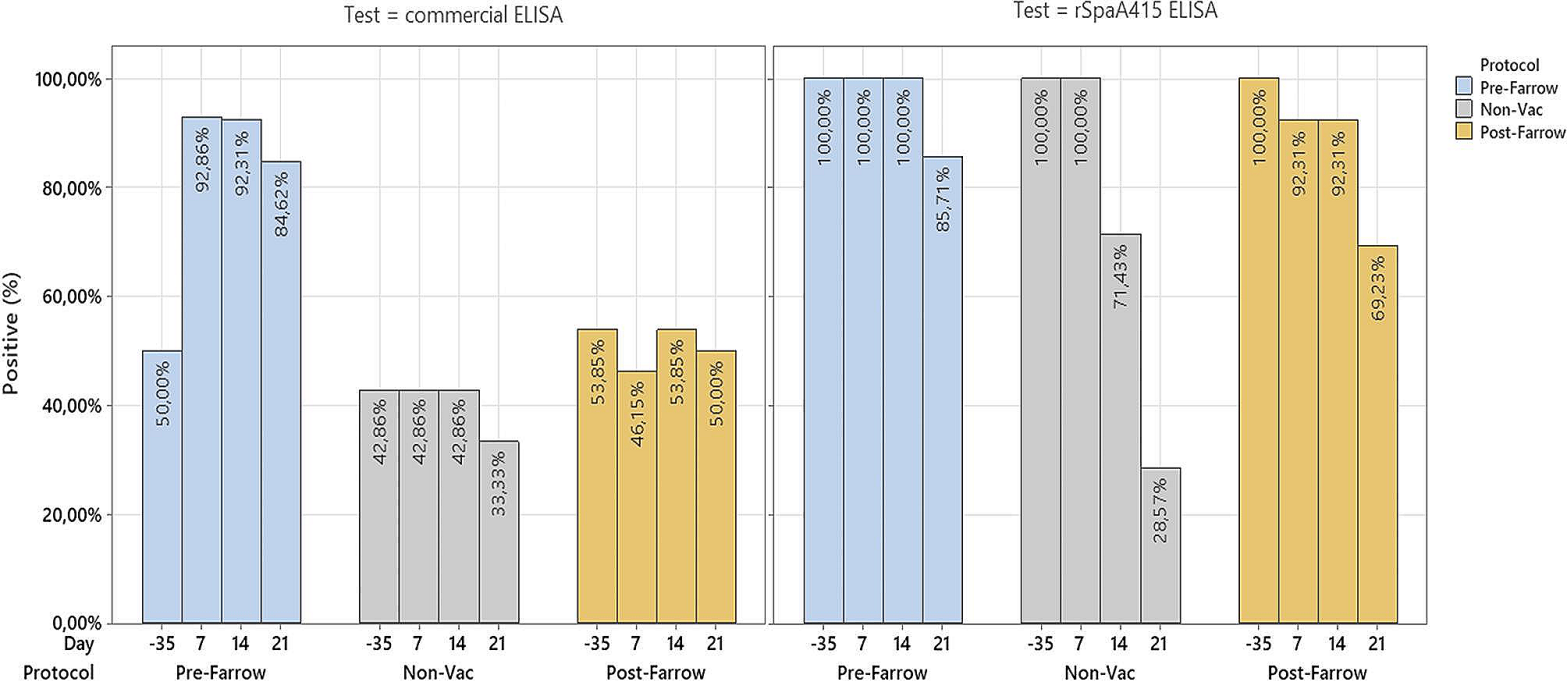
Proportion of *E. rhusiopathiae* seropositive sows within each experimental group from farm B. Seropositive sows in the pre-farrowing group (represented in blue bars) were vaccinated 30 ± 5 days before farrowing (day − 35), non-vaccinated seropositive sows were represented in grey, whereas seropositive sows in the post-farrowing group (represented in light brown) were immunized 10 days after farrowing (day 10). Seroconversion was assessed by means of two different ELISA techniques (Commercial vs. rSpaA415)

The percentage of *E. rhusiopathiae* seropositive sows across the study provided by both ELISA tests, i.e., rSpaA415 and commercial ELISA tests, within each experimental group from farm B is illustrated in Fig. 2. Overall, the rSpaA415 ELISA test detected a higher proportion of seropositive sows compared to the commercial test, regardless of the time point and experimental group considered.

There were no significant differences at the beginning of the study in the percentages of seropositive sows in the different protocols with either of the two tests studied (*p* = 0.28 in the commercial ELISA and *p* = 1 in the rSpaA415 ELISA).

With the commercial ELISA, the percentages of seropositive sows during the lactation period were significantly higher in the PRE-protocol (*p* < 0.001). Remarkably, no significant differences were observed in percentage of seropositive sows from the beginning of the study until the end of the lactation term in the non-vaccinated protocol (*p* = 0.41) and in the post-farrowing protocol (*p* = 0.65).

When we compared with the rSpaA415 ELISA the different time points during the lactation period, we found significant differences in the percentage of seropositive sows among protocols (day 7, 14 and 21 (*p* < 0.001)). The highest percentage of seropositive sows were always found in the pre-farrowing protocol compared to the post-farrowing and non-vaccinated protocols.

A survival time analysis in both types of ELISA was performed in sows from farm B to obtain the days required from the positive to the negative status. In the case of rSpaA415 ELISA, there were no differences in the survival curves when pre-farrowing and post-farrowing vaccination protocols were compared, according to the Log Rank test for survival distribution comparison (*p* = 0.10) [see Additional file 1]. However, when this comparison was performed using the commercial ELISA a trend was observed (*p* = 0.06) in the survival distributions of the vaccination protocols (pre-farrowing vs. post-farrowing) [see Additional file 2]. Additionally, a statistical trend was observed using the Cox-Hazard Proportional Model, which showed that the hazard of testing negative after post-farrowing vaccination with the commercial ELISA was four times higher (4.23 (*p* = 0.073)) than with the pre-farrowing vaccination protocol.

*Objective 2. Evolution of antibody titers*

**Fig. 3:**
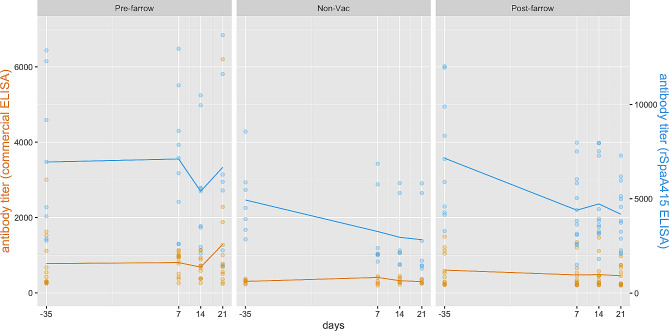
Individual *E. rhusiopathiae* ELISA antibody titer distribution in sows within each group from farm B. Non-vaccinated and vaccinated sows against *E. rhusiopathiae* were included: sows in the pre-farrowing group were vaccinated 30 ± 5 days before farrowing (day − 35), whereas sows in the post-farrowing group were immunized 10 days after farrowing (day 10). Specific antibody titers were determined via two different ELISA tests: Commercial (yellow) and rSpaA415 (blue)

Figure 3 depicts antibody titers in sows from farm B over time using both ELISA tests. According to the graph, the rSpaA415 ELISA determined overall higher antibody titers compared to the commercial test, and a decreasing trend over time was observed in the non-vaccinated and post-farrowing vaccinated groups for both ELISA tests, but especially for rSpaA415 ELISA test.

Given that the level of antibodies was measured for each sow at different time points, and thus longitudinal-type data from both ELISA tests was obtained, a linear mixed model with fixed and random effects was used to study the linear relationship between the level of antibodies in log-scale and potential fixed predictors and random effects (see Statistical analysis Section). Using the commercial ELISA data, no statistically significant differences were observed between vaccination protocols in each considered time point.

However, the overall mean of (log) antibody titers was significantly lower in sows following a post-farrowing vaccine protocol compared to the pre-farrowing vaccinated counterpart (*p* = 0.0278). Using the rSpaA415 ELISA data, the mean of (log) antibody titers of pre-farrowing vs. post-farrowing protocols were significantly different at days 7 (*p* = 0.0062) and 21 (*p* = 0.0026) of the study. Similar to what was observed with the commercial ELISA, the overall mean of (log) antibody titers was significantly lower in non-vaccinated sows (*p* = 0.0056) and sows following a post-farrowing vaccine protocol (*p* = 0.0003) compared to sows vaccinated pre-farrowing. For both ELISA protocols data, there were no differences in the average of the (log) antibody titers between gilts and multiparous sows.

### Serology in piglets

*Objective 1. Seroconversion evolution over time and time-to-negative status*

**Fig. 4:**
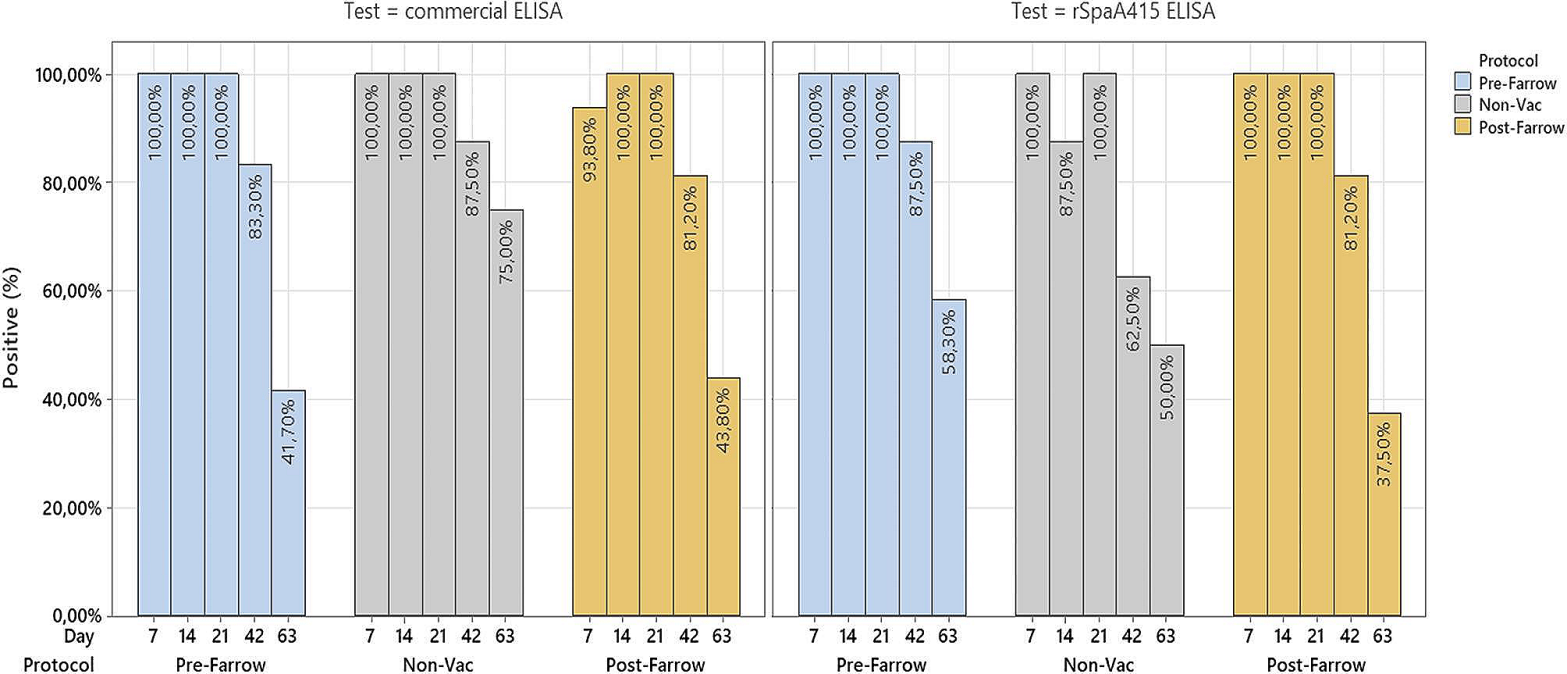
Proportion of *E. rhusiopathiae* seropositive piglets within each experimental group from farm A. Iberian piglets were followed up during the lactation period (days 7, 14, and 21) and after-weaning (days 42 and 63). Seroconversion was assessed by means of two different ELISA techniques (Commercial vs. rSpaA415). The seropositive piglets were represented in blue bars for the pre-farrowing group, in grey bars for the non-vaccinated group and in light brown bars for the post-farrowing group

**Fig. 5:**
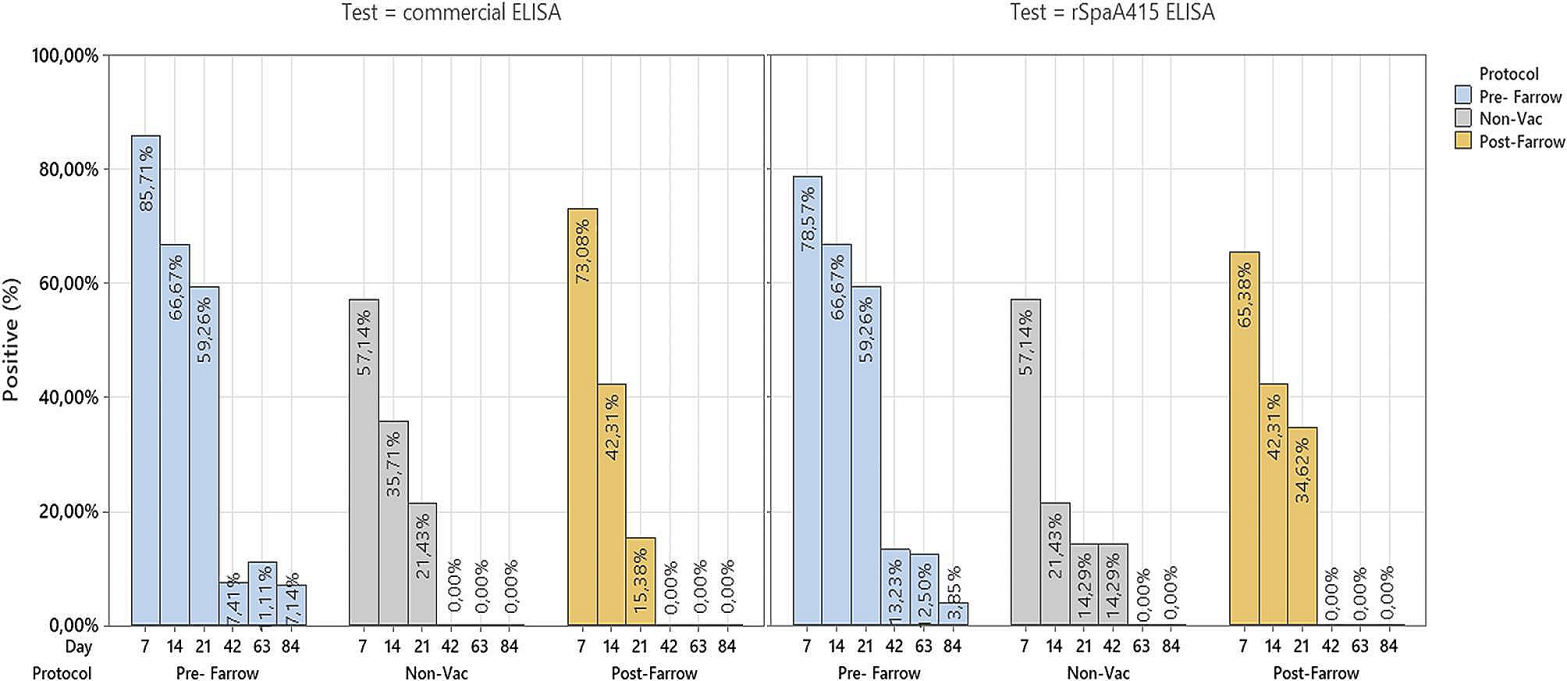
Proportion of *E. rhusiopathiae* seropositive piglets within each experimental group from farm B. Piglets were followed up during the lactation period (days 7, 14, and 21) and after-weaning (days 42, 63 and 84). Seroconversion was assessed by means of two different ELISA techniques (Commercial vs. rSpaA415). The seropositive piglets were represented in blue bars for the pre-farrowing group, in grey bars for the non-vaccinated group and in light brown bars for the post-farrowing group

Distribution of positive ELISA results per vaccination protocol, sampling time point, and ELISA tests are shown in Figs. 4 and 5 for farm A and B, respectively. A higher proportion of seropositive pigs was observed in the Iberian farm A compared to farm B, as indicated by both the rSpaA415 and commercial ELISA tests. In both farms, a certain level of seropositivity to *E. rhusiopathiae* was maintained during the lactation, which reduced significantly during the post-weaning follow-up period.

When we compared the percentages of seropositive piglets in farm A, we found statistical differences between the three protocols with the commercial ELISA test at day 7 (*p* = 0.017) and day 63 (*p* < 0.001). With the rSpaA415 ELISA test, the differences were found at day 14 (*p* < 0.001), 42 (*p* < 0.001) and 63 (*p* = 0.012).

In farm B, with both tests, the percentages of seropositive piglets were significantly higher in the Pre-farrowing group at all time points (lactation and post-weaning period) compared to the post-farrowing and non-vaccinated protocols (*p* < 0.02).

In farm A, there were no differences in the survival distributions of pre-farrowing and post-farrowing vaccinated groups (*p* = 1 and poi = 0.2 for commercial and rSpaA415 ELISA tests, respectively). In farm B, the Log Rank test revealed statistically significant differences in survival distributions between pre-farrowing and post-farrowing vaccinated groups for both the commercial (*p* = 0.005) and rSpaA415 (*p* = 0.01) ELISA tests. Results from the Cox-Hazard Proportional Model for farm B estimated that piglets from post-farrowing vaccinated sows had roughly two times more risk of turning seronegative compared to piglets from pre-farrowing vaccinated sows, and these differences were statistically significant at a 5% significance level using both the commercial and rSpaA415 ELISA tests (2.154 and *p* = 0.008; 2.115 and *p* = 0.011) for the commercial and rSpaA415 ELISA, respectively).

*Objective 2. Evolution of antibodies*

**Fig. 6:**
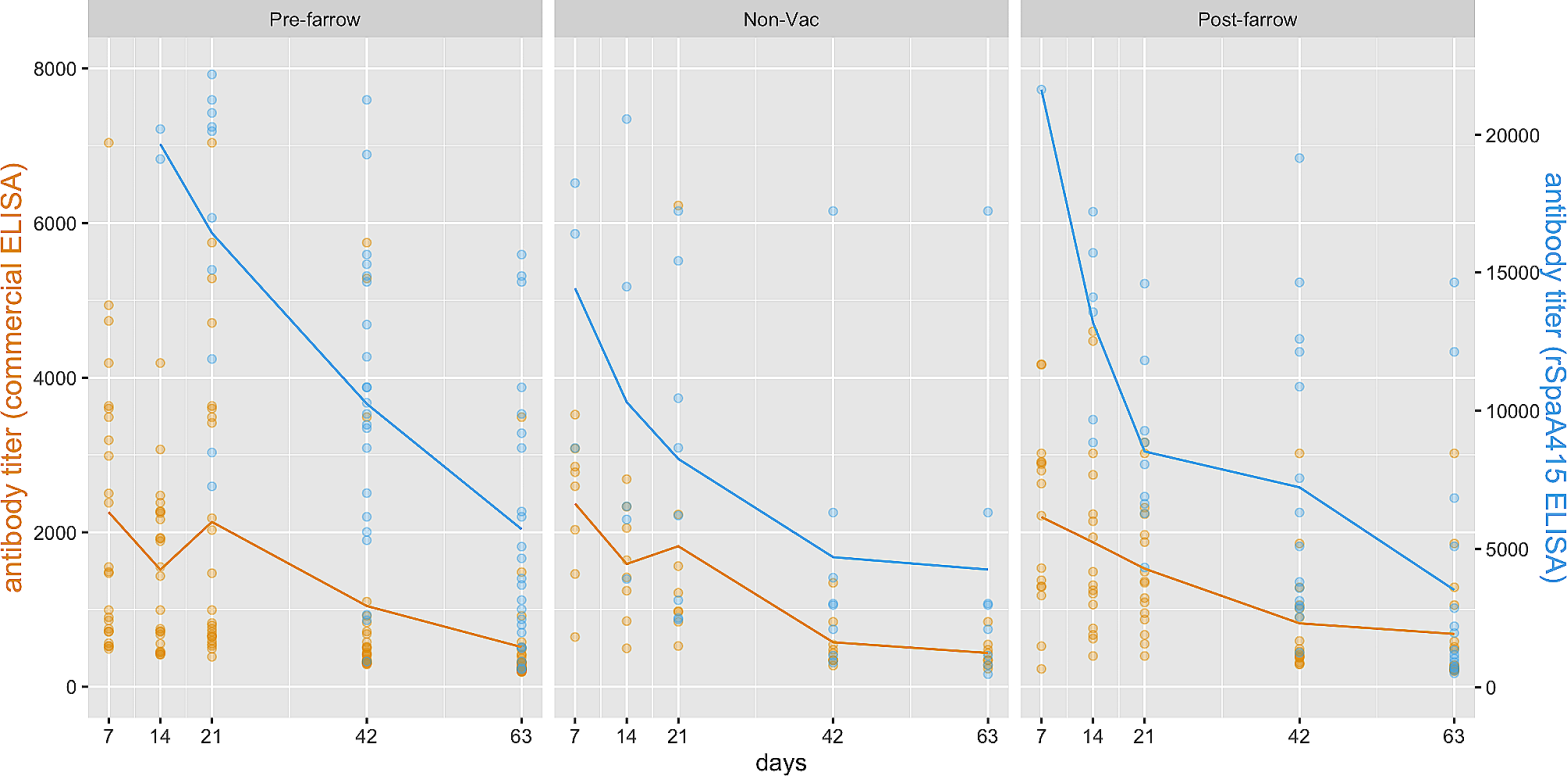
Individual *E. rhusiopathiae* ELISA antibody titer distribution in piglets within each group from farm A. Iberian piglets were followed up during the lactation period (days 7, 14, and 21) and after-weaning (days 42 and 63). Specific antibody titers were determined via two different ELISA tests: Commercial (yellow) and rSpaA415 (blue). Commercial (yellow) and rSpaA415 (blue)

**Fig. 7:**
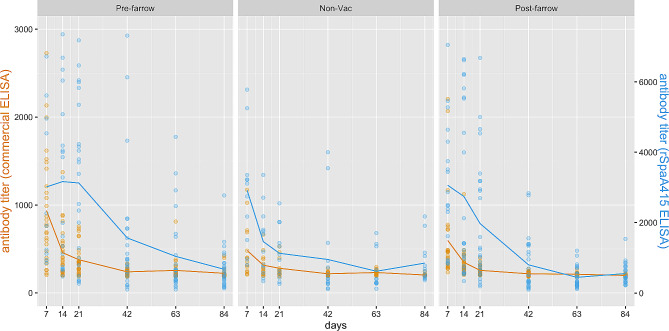
Individual *E. rhusiopathiae* ELISA antibody titer distribution in piglets within each group from farm B. Piglets were followed up during the lactation period (days 7, 14, and 21) and after-weaning (days 42, 63, and 84). Specific antibody titers were determined via two different ELISA tests: Commercial (yellow) and rSpaA415 (blue)

Figures 6 and 7 show the evolution of antibody titers for farms A and B, respectively, based on the vaccination protocols and ELISA tests. Higher antibody titers were determined by the rSpaA415 ELISA compared to the commercial test, and a steep decay in antibody levels, especially with the rSpaA415 ELISA, was generally observed during the follow-up period, regardless of the experimental group.

The overall mean of (log) antibody titers in piglets from the post-farrowing vaccination protocol was significantly lower than in piglets from the pre-farrowing vaccination protocol in farm A (*p* = 0.0059; rSpaA415 ELISA) and farm B (*p* = 0.0168 and *p* = 0.0098 for the commercial and rSpaA415 ELISA data, respectively).

In farm A, the linear mixed model revealed no statistically significant differences between pre- and post-farrowing vaccination protocols in any of the time points evaluated for both the commercial and rSpaA415 ELISA tests. However, when considering results from the rSpaA415 ELISA test, statistically significant differences in the mean of (log) antibody titers between pre-farrowing and non-vaccinated groups were found at days 14 (*p* = 0.0403), 21 (*p* < 0.0001) and 42 (*p* = 0.0327). In addition, there were statistically significant differences in the mean of (log) antibody titers between different parity sows for both rSpaA415 and commercial ELISA tests; the mean (log) antibody titers was significantly lower in gilts than in multiparous sows (*p* = 0.0015 and *p* = 0.0011 for the commercial and rSpaA415 ELISA tests, respectively).

On the other hand, for farm B, no statistically significant differences were observed between pre- and post-farrowing vaccination protocols in any of the time points considered when evaluated with the rSpaA415 ELISA data. Nevertheless, significant differences in the mean of (log) antibody titers were found between pre-farrowing and non-vaccinated groups (*p* = 0.0016) and pre- vs. post-farrowing vaccination protocols on day 7 (*p* = 0.0015) when considering results from the commercial ELISA test. For farm B, there were no statistically significant differences in the mean of log antibodies between gilts and multiparous neither for rSpaA415 ELISA data nor for commercial ELISA data.

*Objective 3. Correlation between E. rhusiopathiae ELISA titers in sows and their offspring, and the agreement between the ELISA tests.*

### Correlation between sows and piglets serologies

**Fig. 8:**
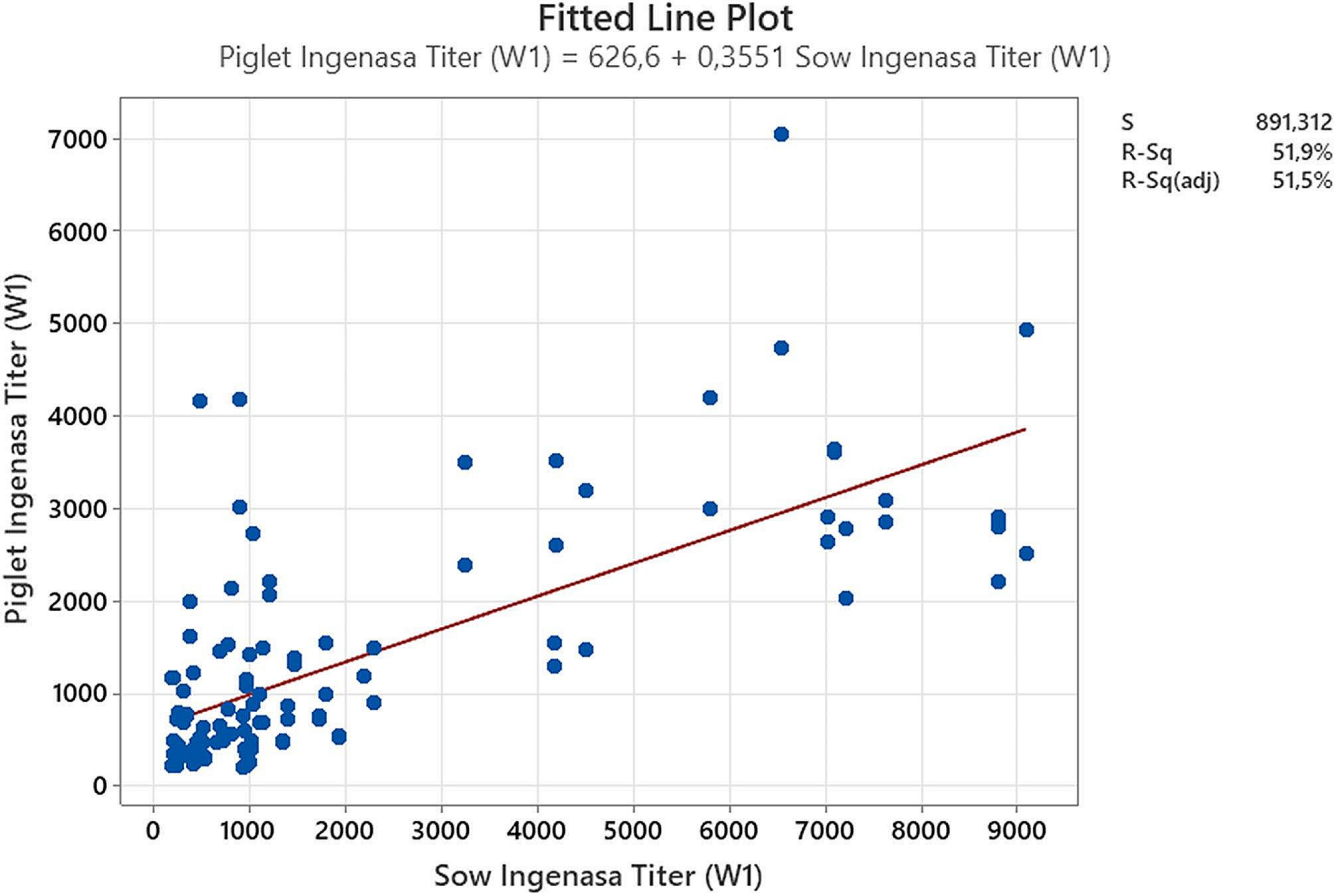
Correspondence of *Erysipelothrix rhusiopathiae* antibodies titers obtained by Ingenasa ELISA test between sows and their piglets one week after farrowing. Blue dots reflect each corresponding titer between sows and their piglets. The data correlation was analyzed by linear regression

**Fig. 9:**
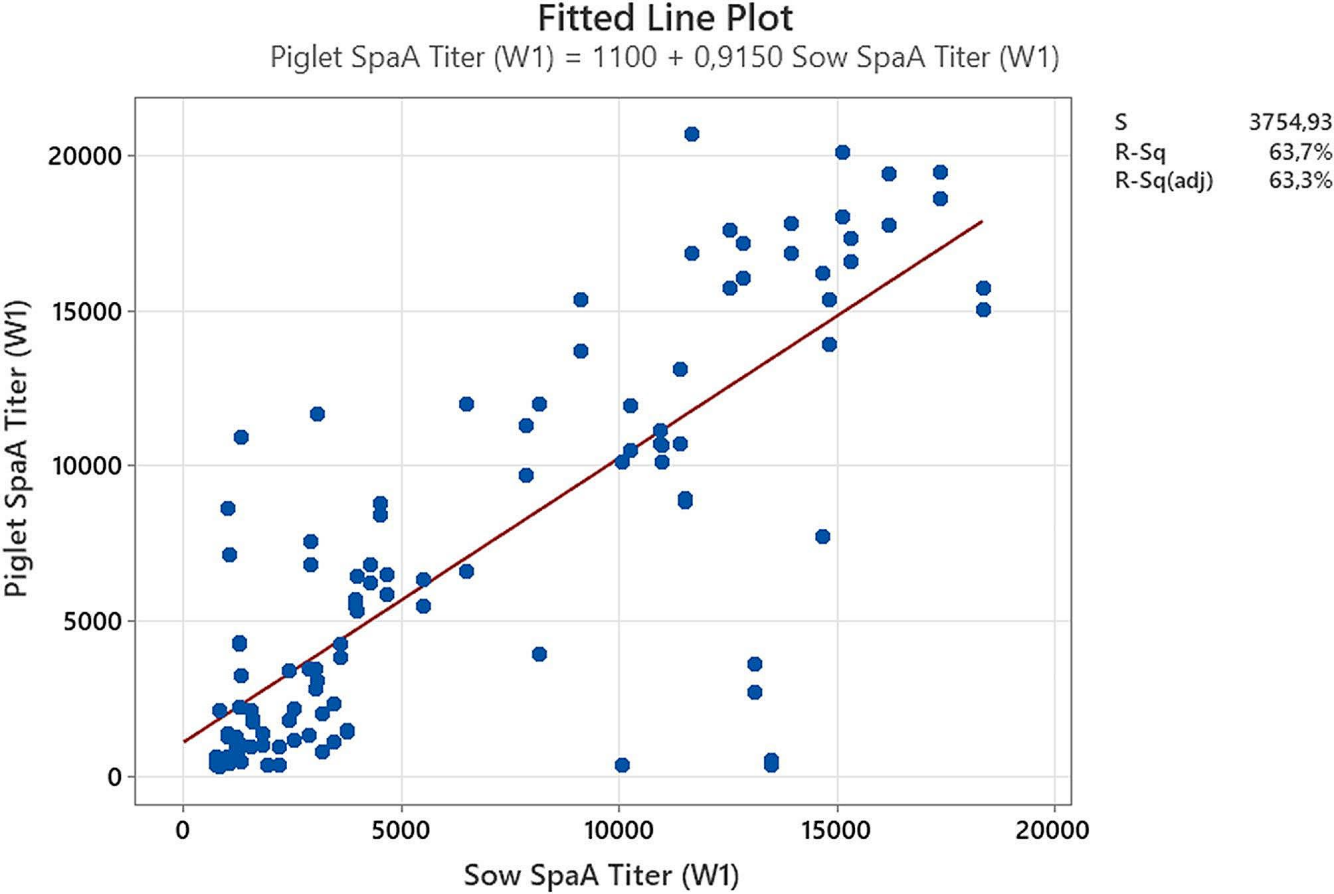
Correspondence of Erysipelothrix rhusiopathiae antibodies titers obtained by rSpaA415 ELISA test between sows and their piglets one week after farrowing. Blue dots reflect each corresponding titer between sows and their piglets. The data correlation was analyzed by linear regression

Upon analysis of the aggregated titers (from the two farms) of one week post farrowing sows and their 1-week-old piglets, a statistical correlation was identified in both tests. The R^2^ and *P* values for the Ingenasa test were 0.52 and < 0.001 (Fig. 8) respectively whereas R^2^ and *P* values for the rSpaA415 ELISA test were 0.64 and < 0.001 (Fig. 9).

### Agreement analysis

A total of 811 serological results were used to perform the agreement test between the two ELISA tests utilized in this study. The results demonstrated a substantial agreement (Cohen’s Kappa of 0.68 ± 0.03; *p* < 0.001) between the commercial ELISA and rSpaA415 ELISA test.

## Discussion

A protective role of the specific antibodies in erysipelas has long been suggested given that immunization with bacterins is widely used for disease control in pigs. While vaccination is standard in most pig breeding herds, growing pigs are not commonly vaccinated against erysipelas as they are expected to have MDA when leaving the breeding farm [11]. Nevertheless, vaccination approaches in sows to maximize *E. rhusiopathiae*-MDA in piglets have not been investigated. In this light, the work presented herein aimed to evaluate potential differences in antibody levels in sows and their offspring when two different erysipelas vaccination protocols (pre-farrowing vs. post-farrowing) were applied in conventional Large white-Landrance and Iberian breeding pig farms.

Overall, the results indicate that vaccination timing in sows enhanced maternal antibody (MDA) levels in pigs in a global environment where the antibiotic usage is been reducing by the industry.

In this study, serum samples were tested using two indirect ELISA tests that detect *E. rhusiopathiae*–specific antibodies: a commercially available ELISA kit (Ingenasa) and an ELISA based on a recombinant SpaA protein (rSpaA415) [17]. These two kits have been previously compared, with the total sensitivity rate on experimentally infected pigs being 26.4% and 73.6% in commercial and rSpaA415 ELISA tests, respectively [24]. In contrast to the findings of Giménez-Lirola et al., which indicated a slight degree of concordance (Kappa statistics = 0.15 ± 0.05), our results demonstrate a substantial degree of agreement between the two tests (Kappa statistics = 0.68 ± 0.03). In line with the previous research, the observed sensitivity to determine the immunological status of sows and piglets in the current study was higher for the rSpaA415 ELISA compared to the commercial one, as determined by an overall higher proportion of seropositive animals regardless of the group and time point of evaluation. However, because the current study was conducted on herds with unknown disease status, further research should investigate if similar results can be obtained using confirmed non-infected/infected pigs.

The absorption of colostral immunoglobulins (Igs) in the piglet is nonspecific, and the concentrations of Igs in the piglet circulation bear a resemblance to those of the sow [25]. In our study we have identified for the first time a correlation between *E. rhusiopathiae* titers among sows and their one-week old piglets using two ELISA tests. Hence, sow serum Igs may be helpful to predict the piglets’ specific antibody concentrations. In fact, using the Ingenasa ELISA kit for *E. rhusiopathiae*–antibodies detection, sow colostrum and sow serum optical densities (OD) were used to predict piglets OD [18]. The correlation (R^2^) between the ELISA titers of sows and their offspring (0.52 for the commercial ELISA and 0.64 for rSpaA415 ELISA) has also been investigated using different ELISA tests and diseases with similar results. For instance, Boonsoongnern et al., obtained a correlation (Pearson’s *r* = 0.74) in serum titers of porcine epidemic diarrhea from sows at 7 days before farrowing and their 7-day-old piglets [26]. Regarding, Porcine Reproductive and Respiratory Syndrome one of the costliest diseases in porcine a correlation of neutralizing antibodies between sows and their 2 weeks old piglets was also found (R^2^ = 0.3377; *p* < 0.0001) [27].

In the Iberian swine production (farm A), antibody levels in sows were higher compared to conventional white sows (farm B), and all the Iberian sows remained seropositive throughout the study period. This latter could be explained by a higher exposure to *E. rhusiopathiae*, which is common in outdoor organic farming [11]. Furthermore, investigations indicate that during a clinical outbreak of swine erysipelas, Erysipelothrix spp. can be isolated from a variety of environmental samples like manure, feed, nipples drinker and the walls [28]. Also, acclimation against erysipelas using vaccination was more intensive in gilts from farm A than from farm B, providing a higher immunological baseline against E. rhusiopathiae. Consequently, we could not evaluate whether the vaccination protocol exerts a certain effect on the Iberian sows’ seroconversion pattern during the period evaluated in farm A.

For farm B, the overall mean antibody titer was significantly higher in sows vaccinated pre-farrowing than either non-vaccinated sows or sows vaccinated post-farrowing, coinciding with a higher percentage of seropositive sows from the first week post-farrowing onwards. Furthermore, the post-farrowing vaccination protocol exhibited a tendency to increase the risk of becoming seronegative during lactation by four times compared to the pre-farrowing vaccination protocol, as indicated by the Ingenasa ELISA data. Our results thus indicated that sows vaccinated prior to farrowing reached higher levels of Igs at lactation than sows vaccinated 10 days after farrowing. Indeed, pre-farrowing vaccination protocols are commonly used in the pig veterinary practice to improve colostral immunity and better control other swine diseases caused by bacteria, such as progressive atrophic rhinitis, or *Streptococcus suis*, *Escherichia coli* and clostridial infections [29–32]. In addition, it should be mentioned though that the vaccines employed in the present study (i.e., Ruvax® and Eryseng®) are commercial monovalent vaccines, but the most used standard sow vaccination protocols at every breeding cycle during lactation term are mostly applied with bivalent commercial vaccines (Parvovirus and Erisipela) or trivalent commercial vaccines (Parvovirus, Erisipela and Leptospira).

Colostral antibodies protect piglets through early exposure periods to many of the economically important disease agents circulating in pig farms [31]. Hence, it is interesting to investigate whether vaccinating sows during gestation could maximize piglet humoral immunity against *E. rhusiopathiae* at weaning, in the nursery and finishing units. In agreement with the obtained serological results in sows, higher antibody titers, and thus a higher proportion of seropositive pigs, was documented in the Iberian farm A compared to farm B, as indicated by both ELISA tests. However, a remarkable decrease in mean antibody titers was observed in both vaccination protocol scenarios during lactation. Nevertheless, all tested piglets from farm A were seropositive at weaning, and most of them became seronegative between sampling days 42 and 63 post-farrowing regardless of the treatment group they belonged to. In farm B, most piglets became seronegative by day 42 post-farrowing, except a minority from the pre-farrowing vaccination treatment that remained seropositive up to day 84 post-farrowing. Overall, the mean antibody titer in piglets from the post-farrowing vaccination protocol was significantly lower than in piglets from the pre-farrowing vaccination protocol in both farms. In addition, piglets in farm B from sows vaccinated post-farrowing had two times more risk of turning seronegative than piglets from sows vaccinated pre-farrowing. However, even though the pre-farrowing gilt/sow vaccination resulted in increased antibody responses, the MDA duration did not last long enough to cover the post-weaned period evaluated completely. Whether the cellular immune response would follow a similar pattern is unknown and deserve to be investigated to warrant optimized vaccination strategies against the disease. In any case, MDA dynamics need to be considered if growing-finishing pigs are vaccinated, as several studies have shown that vaccination against *E. rhusiopathiae* is only protective when pigs are vaccinated after MDA decrease [15, 16].

The current welfare rules, the rise of outdoor and/or organic swine farms, and the systematic reduction in the use of antimicrobials in swine production [30] have influenced changes in the dynamics of *E. rhusiopathiae* infection and the appearance of disease [11]. To better control erysipelas, it is important to optimize vaccination protocols and achieve high disease-specific antibody levels in the sow colostrum. In this line, our results revealed that higher *E. rhusiopathiae* antibody titers in sows and their offspring and lower risks to become seronegative during lactation or peri-weaning periods could be achieved following a pre-farrowing vaccination protocol.

## Conclusion

The present results highlight the importance of optimizing vaccination protocols to achieve optimal levels of immunity in sows at parturition. This is due to the correlation between titers found in sows and their piglets which has the potential to enhance their humoral disease-specific immunity. While most of the currently available vaccines against *E. rhusiopathiae* are administered after farrowing, the results obtained herein emphasize that pre-farrowing vaccination strategies could be an alternative approach to increase the humoral immune response in sows and piglets and achieve a higher and longer protection against erysipelas in weaned pigs.

## Electronic supplementary material

Below is the link to the electronic supplementary material.

**Additional File 1.** Survival time analysis to study the time from positive to negative status using rSpaA415 ELISA in sows from Farm B, between pre-farrowing (red) and post-farrowing (blue) vaccination protocol. Sows were followed up at day 35 before farrowing (-35) and during the lactation period (days 7, 14, and 21).

**Additional File 2.** Survival time analysis to study the time from positive to negative status using commercial ELISA in sows from Farm B, between pre-farrowing (red) and post-farrowing (blue) vaccination protocol. Sows were followed up at day 35 before farrowing (d-35) and during the lactation period (days 7, 14, and 21).

## Data Availability

All relevant data are within the manuscript and its Additional Information files.

## Abbreviations

MDA: Maternally derived antibodies
E. rhusiopathiae: Erysipelothrix rhusiopathiae
ELISA: Enzyme-linked immunosorbent assay
Igs: Immunoglobulins
OD: Optical densities
I.M.: Intramuscular
LMM: Linear mixed model
