# Supplementary material for: A field comparison study of two vaccine protocols against Erysipelothrix rhusiopathiae in two types of swine breeds in Spain
Source: BMC Vet Res. 2024 Oct 11;20:461. doi: 10.1186/s12917-024-04065-0 (PMC11468219; doi:10.1186/s12917-024-04065-0)
Supplement: Supplementary file 1 — Additional File 1. Survival time analysis to study the time from positive to negative status using rSpaA415 ELISA in sows from Farm B, between pre-farrowing (red) and post-farrowing (blue) vaccination protocol. Sows were followed up at day 35 before farrowing (-35) and during the lactation period (days 7, 14, and 21). [file 12917_2024_4065_MOESM1_ESM.docx]

**Supplementary Information**


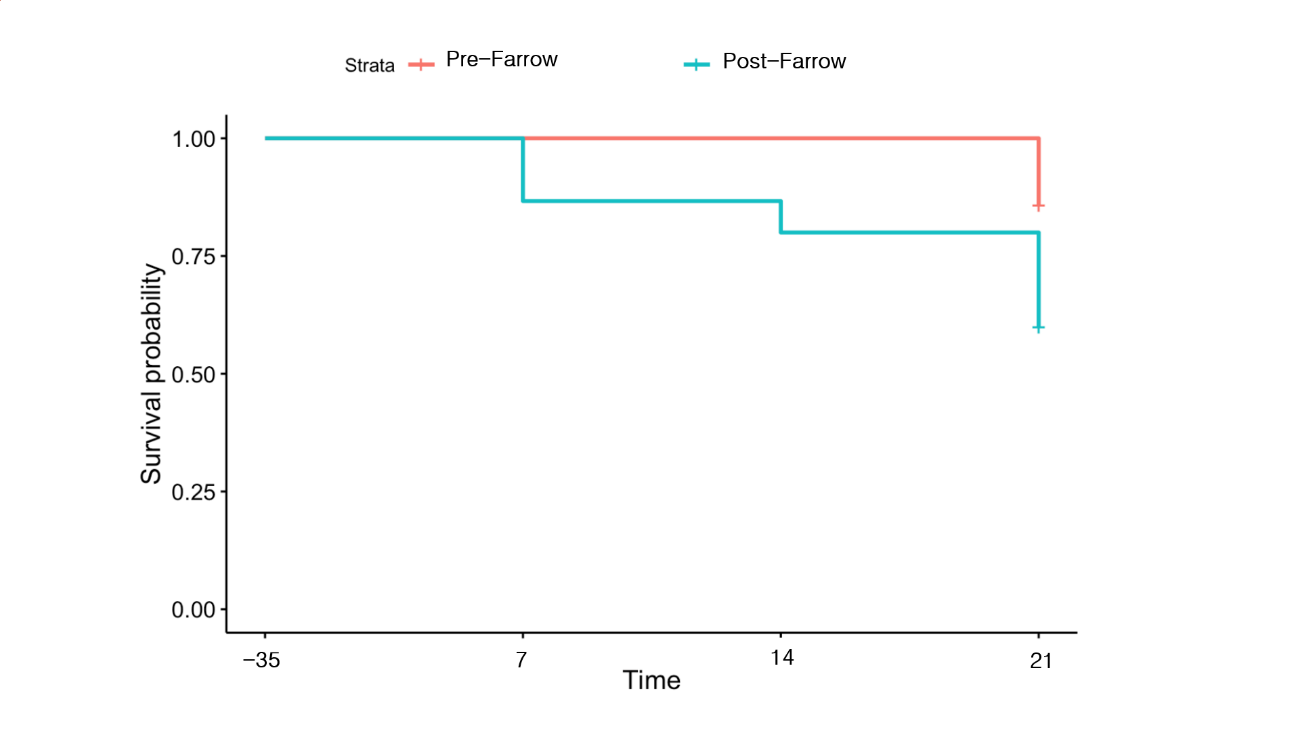


**Additional file 1:** Survival time analysis in sows for SpaA protein ELISA (rSpaA415).
