# Supplementary material for: A field comparison study of two vaccine protocols against Erysipelothrix rhusiopathiae in two types of swine breeds in Spain
Source: BMC Vet Res. 2024 Oct 11;20:461. doi: 10.1186/s12917-024-04065-0 (PMC11468219; doi:10.1186/s12917-024-04065-0)
Supplement: Supplementary file 2 — Additional File 2. Survival time analysis to study the time from positive to negative status using commercial ELISA in sows from Farm B, between pre-farrowing (red) and post-farrowing (blue) vaccination protocol. Sows were followed up at day 35 before farrowing (d-35) and during the lactation period (days 7, 14, and 21). [file 12917_2024_4065_MOESM2_ESM.docx]

**Supplementary Information**


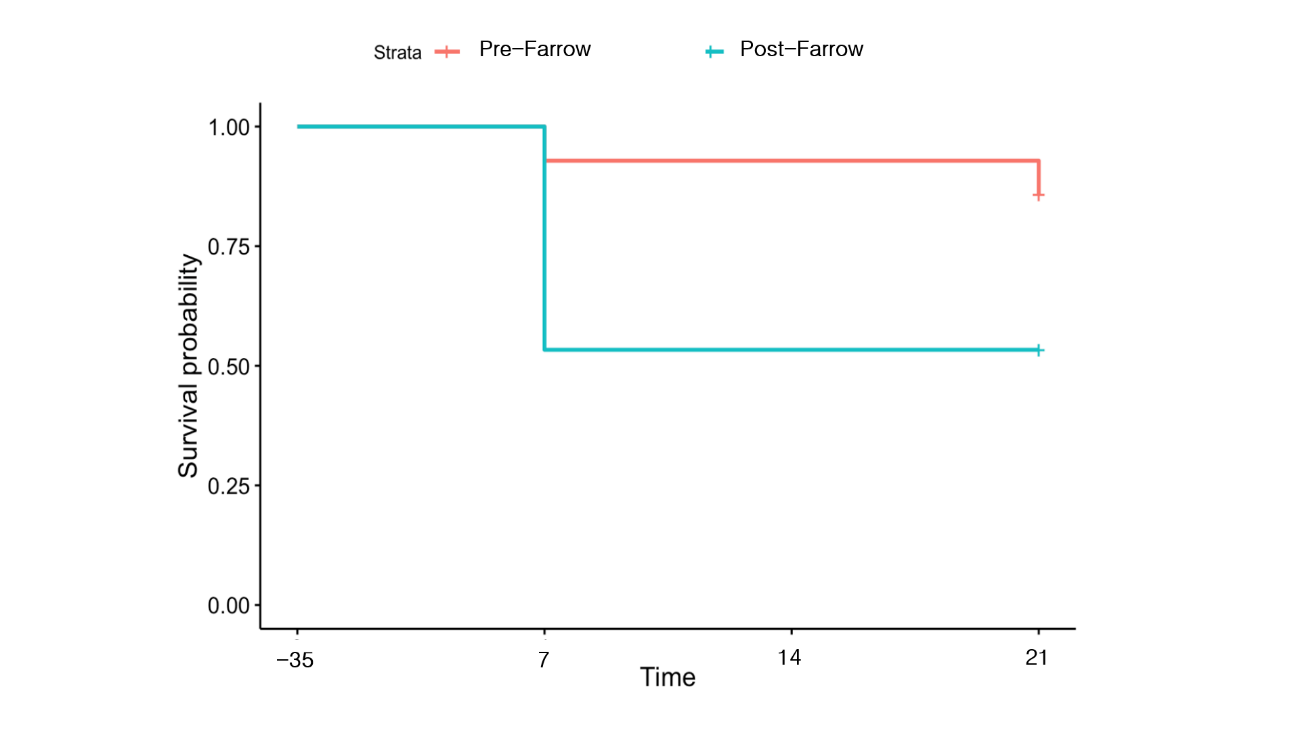


**Additional File 2:** Survival time analysis in sows for commercial ELISA (Ingezim®).
